# Construction of Tandem Multimers with Different Combinatorial Forms of BmSPI38 and BmSPI39 and Analysis of Their Expression and Activity in *Escherichia coli*

**DOI:** 10.3390/ijms26051788

**Published:** 2025-02-20

**Authors:** Zhaofeng Zhang, Youshan Li, Xi Yang, Changqing Chen, Shuai Ru, Jie Jiang, Wenyao Cai, Jiyu Li, Juanle Du, Dejue Qiao

**Affiliations:** 1 College of Biological Science and Engineering, Shaanxi University of Technology, Hanzhong 723001, China; zhang_zhaofeng2021@163.com (Z.Z.); yx1910928061@163.com (X.Y.); 2 Qinba Mountain Area Collaborative Innovation Center of Bioresources Comprehensive Development, Hanzhong 723001, China; chen_changq@163.com (C.C.); ru_shuai2023@163.com (S.R.); 3 Qinba State Key Laboratory of Biological Resources and Ecological Environment (Incubation), Shaanxi University of Technology, Hanzhong 723001, China; jiang_jie907@163.com (J.J.); 18283324539@163.com (W.C.); 13327691302@163.com (J.L.); 4 Shaanxi Province Key Laboratory of Bio-Resources, Hanzhong 723001, China; dujuanle9@163.com (J.D.); q1303296732@163.com (D.Q.)

**Keywords:** *Bombyx mori*, protease inhibitor, heterotypic tandem multimer, *Escherichia coli*, heterologous expression, inhibitory activity

## Abstract

It was found that the serine protease inhibitors BmSPI38 and BmSPI39 in silkworm can strongly inhibit the activity of porcine pancreatic elastase, which has potential applicational value in the drug research and development of lung diseases, inflammatory diseases, and skin aging caused by the excessive release of elastase. Previous studies have shown that homotypic multimers obtained by tandem expression can significantly enhance the antifungal activity and structural homogeneity of BmSPI38 and BmSPI39, while the effect of the tandem expression of these two inhibitors, with different combinations, on the total activity and expression levels of multimers remains unclear. The aim of this study is to explore whether it is possible to obtain the combination of BmSPI38 and BmSPI39 with strong total expression activity by protein engineering. In this study, 40 tandem multimer expression vectors with different combinatorial forms of BmSPI38 and BmSPI39 were constructed by the isocaudomer method, and recombinant proteins were obtained by the prokaryotic expression system. The target proteins were separated by SDS-PAGE to analyze the expression levels of multimer proteins with different combinatorial forms. The total activity of the recombinant expression products with different tandem forms was investigated using the in-gel activity staining technique of protease inhibitors. The SDS-PAGE results show that the expression levels of tandem multimers containing the BmSPI39 module at the carboxyl terminus were generally higher in the *Escherichia coli* supernatant than that of the tandem multimers containing the BmSPI38 module at the carboxyl terminus. The activity staining results indicate that compared with BmSPI38 and BmSPI39 homotypic multimers, the total activity of some recombinant expression products with different tandem forms was stronger. Furthermore, the total activity level was relatively higher when the carboxyl terminus of the multimer was a BmSPI39 module, such as the tandem dimers SPIAB and SPIaB and the tandem trimers SPIabB, SPIaaB, and SPIbaB. In this study, the expression of tandem fusion proteins with different combinations of the silkworm protease inhibitors BmSPI38 and BmSPI39 in *E*. *coli* was successfully achieved. It was confirmed that the tandem of different combinatorial forms, based on protein engineering, was an effective way to enhance the total activity of the fusion proteins of BmSPI38 and BmSPI39 and to improve their expression levels. Additionally, a number of multimer proteins with strong total activity and high exogenous expression levels were also screened, for example, SPIbaA, SPIbbA, SPIbbB, SPIabB, SPIaaB, and SPIbaB. This study not only lays the foundation for the exogenous production and development of BmSPI38 and BmSPI39 but also provides a reference for the construction of tandem and multimerization exploration of other protease inhibitors.

## 1. Introduction

Protease inhibitors are pivotal regulators of protease catalytic activity in vivo, which can bind to proteases, inhibiting their physiological functions and thus terminating unnecessary proteolysis processes [1]. They play an important role in a variety of physiological processes, including digestion, coagulation, cell migration, angiogenesis, and inflammation. In view of the importance of protease inhibitors, related research reports have gradually increased in recent years. Researchers have concentrated their efforts on the discovery of new inhibitors, the study of inhibitory activity and characteristics, and even their design through artificial synthesis technology [2,3,4]. Relevant research results have had an important impact in the fields of medicine, agriculture, and the ecological environment [5,6,7,8].

As an important economic insect, silkworm has become an ideal model in the fields of biochemistry, genetics, and genomics [9,10,11]. In contrast to mammals, insects lack lymphocytes and immunoglobulins, while serine protease inhibitors are believed to play a crucial role in insect immunity [12,13,14]. In the initial stages of this investigation, our team identified 80 serine protease inhibitor (SPI) genes from the silkworm genome. A total of 10 SPI domains were identified from these serine protease inhibitors, such as Serpin, Kunitz, Trypsin inhibitor-like cysteine-rich (TIL) domains, and Kazal [15]. BmSPI38 and BmSPI39 have a unique TIL domain, which can inhibit the activity of CDEP-1, a virulence protease of *Beauveria bassiana*, thus enhancing the antifungal ability of silkworm [16,17]. Silkworm contains abundant protease inhibitors. TIL family protease inhibitors represented by BmSPI39 can enter the cocoon layer during silk secretion, endowing the cocoon with powerful antimicrobial protease hydrolysis properties and providing long-term protection for the development of silkworm pupae in the cocoon [18,19]. It has been demonstrated that the recombinant proteins BmSPI38 and BmSPI39 are prone to spontaneous multimerization, forming dimers, trimers, and tetramers. In silkworm tissues, these two proteins mainly exist and exert biological functions in the form of tetramers [20]. By analyzing the expression and activity of the BmSPI38 and BmSPI39 of homologous multimers, it was found that homologous tandem expression can improve their antifungal activity and structural homogeneity [21,22]. In view of this, is it possible to obtain multimer proteins with stronger total activity, higher expression levels, and lower production costs by different combinations of BmSPI38 and BmSPI39 in tandem based on protein engineering, thereby achieving the effect of “1 + 1 > 2”? This issue requires further exploration.

In this study, we planned to construct tandem dimer, trimer, and tetramer expression vectors with different combinatorial forms of BmSPI38 and BmSPI39, obtaining recombinant multimer proteins by a prokaryotic expression system. The tandem multimers with strong total activity and high exogenous expression levels were screened using the in-gel activity staining technique of protease inhibitors. This study not only lays the foundation for the large-scale production of BmSPI38 and BmSPI39 and their development and application in the field of medicine but also helps to deepen the understanding of their multimerization mechanisms. It can also provide a reference for the tandem construction and multimerization exploration of other protease inhibitors.

## 2. Results

### 2.1. Design and Construction of Tandem Multimer Expression Vectors with Different Combinatorial Forms of BmSPI38 and BmSPI39

**Figure 1:**
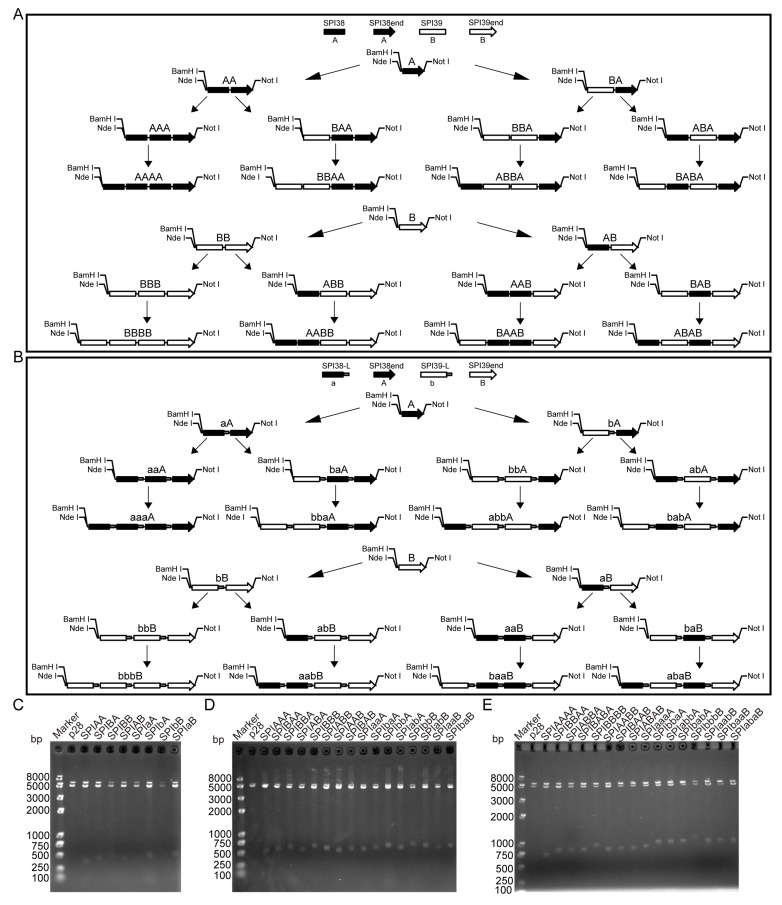
Design and construction of tandem multimer expression vectors with different combinatorial forms of BmSPI38 and BmSPI39. (**A**) Schematic diagram of the construction of expression vectors for tandem multimers of BmSPI38 and BmSPI39 in different combinatorial forms without the flexible linker sequence. “SPI38” represents the coding sequence of the BmSPI38 protein module, and “SPI38end” represents the coding sequence of the BmSPI38 protein module with a termination codon, both of which are referred to by the letter “A”. “SPI39” represents the coding sequence of the BmSPI39 protein module, and “SPI39end” represents the coding sequence of the BmSPI39 protein module with a termination codon, both of which are referred to by the letter “B”. (**B**) Schematic diagram of the construction of expression vectors for tandem multimers of BmSPI38 and BmSPI39 in different combinatorial forms with the flexible linker sequence added. “SPI38-L” and “SPI39-L”, respectively, represent the coding sequences of the BmSPI38 and BmSPI39 protein modules with a flexible linker, denoted here by the letters “a” and “b”, respectively. (**C**) Analysis of double-enzyme digestion products of tandem dimer expression plasmids by agarose gel electrophoresis. (**D**) Analysis of double-enzyme digestion products of tandem trimer expression plasmids by agarose gel electrophoresis. (**E**) Analysis of double-enzyme digestion products of tandem tetramer expression plasmids by agarose gel electrophoresis. The p28 plasmid is a derivative expression plasmid of pET28b. The bands of approximately 5000 bp are linearized vector fragments generated by double-enzyme digestion.

In order to obtain active tandem multimer proteins with different combinatorial forms of BmSPI38 and BmSPI39, two strategies for the construction of expression vectors were designed. The first strategy is to add no flexible linker sequence between fusion proteins (Figure 1A). Firstly, the BmSPI38 coding frame sequence “A”, which removes the signal peptide and carries a termination codon, was designed at the 3′ end of the fusion gene expression vector. Then, the 5′ end of the vector was ligated with either the signal-peptide-removed BmSPI38 coding frame sequence “A” or the signal-peptide-removed BmSPI39 coding frame sequence “B” by using the isocaudomer method, resulting in the recombinant dimer expression plasmids *SPIAA* and *SPIBA*. Subsequently, “A” or “B” was connected to the 5′ end of *SPIAA* and *SPIBA*, respectively, so as to obtain recombinant trimer expression plasmids *SPIAAA*, *SPIBAA*, *SPIBBA*, and *SPIABA*. Finally, tandem tetramer expression plasmids (*SPIAAAA*, *SPIBBAA*, *SPIABBA*, and *SPIBABA*) were obtained by connecting “A” at the 5′ end of *SPIAAA*, “B” at the 5′ end of *SPIBAA*, “A” at the 5′ end of *SPIBBA*, and “B” at the 5′ end of *SPIABA*. Similarly, the BmSPI39 coding frame sequence “B”, removing the signal peptide and with a termination codon, was designed at the 3′ end of the fusion gene expression vector, and the BmSPI39 coding frame sequence “B”, without the signal peptide, or the BmSPI38 coding frame sequence “A”, without the signal peptide, was ligated to its 5′ end, thus obtaining the recombinant dimer expression plasmids *SPIBB* and *SPIAB*. Then, tandem trimer expression plasmids (*SPIBBB*, *SPIABB*, *SPIAAB*, and *SPIBAB*) and tandem tetramer expression plasmids (*SPIBBBB*, *SPIAABB*, *SPIBAAB*, and *SPIABAB*) were constructed in turn by the isocaudomer method. The second strategy is to add a flexible linker sequence between the fusion proteins (Figure 1B). The flexible linker is an amino acid chain that connects two fusion proteins. It has a certain amount of flexibility and helps the protein to fold correctly during expression, allowing the proteins on both sides to perform their independent functions [23,24]. The glycine-rich flexible linker sequence (GGGGS)_n_ is a linker commonly used to connect different fusion proteins. The linker sequence used here is “GGGGSGGGGSGGGGS”, and its corresponding encoding sequence is “GGCGGTGGTGGCTCAGGCGGTTGGCTCAGGCGGTGGTGGCTCA”. Recombinant dimer expression plasmids with flexible linkers (*SPIaA*, *SPIbA*, *SPIbB*, and *SPIaB*); recombinant trimer expression plasmids with flexible linkers (*SPIaaA*, *SPIbaA*, *SPIbbA*, *SPIabA*, *SPIbbB*, *SPIabB*, *SPIaaB*, and *SPIbaB*); and recombinant tetramer expression plasmids with flexible linkers (*SPIaaaA*, *SPIbbaA*, *SPIabbA*, *SPIbabA*, *SPIbbbB*, *SPIaabB*, *SPIbaaB*, and *SPIabaB*) were constructed using the method described above.

Double-restriction-enzyme digestion was performed using *Nde* I and *Not* I, and 1.5% agarose gel electrophoresis was used to detect whether tandem multimer expression plasmids had been successfully constructed. The results show that DNA fragments, ranging in size from 250 bp to 500 bp, were detected in the double-digestion products of the tandem dimer expression vectors *SPIAA*, *SPIBA*, *SPIBB*, *SPIAB*, *SPIaA*, *SPIbA*, *SPIbB*, and *SPIaB* (Figure 1C). DNA fragments, with sizes ranging from 500 bp to 750 bp, were detected in the double-digestion products of the tandem trimer expression vectors *SPIAAA*, *SPIBAA*, *SPIBBA*, *SPIABA*, *SPIBBB*, *SPIABB*, *SPIAAB*, *SPIBAB*, *SPIaaA*, *SPIbaA*, *SPIbbA*, *SPIabA*, *SPIbbB*, *SPIabB*, *SPIaaB*, and *SPIbaB* (Figure 1D). Similarly, DNA fragments, with a size between 750 bp and 1000 bp, were detected in the double-enzyme digestion products of the tandem tetramer expression vectors *SPIAAAA*, *SPIBBAA*, *SPIABBA*, *SPIBABA*, *SPIBBBB*, *SPIAABB*, *SPIBAAB*, *SPIABAB*, *SPIaaaA*, *SPIbbaA*, *SPIabbA*, *SPIbabA*, *SPIbbbB*, *SPIaabB*, *SPIbaaB*, and *SPIabaB* (Figure 1E). The size of the enzyme digestion products of the above expression vectors was consistent with the expected size. After further DNA sequencing verification, the above 40 recombinant expression vectors were successfully constructed.

### 2.2. Prokaryotic Expression of Tandem Multimers with Different Combinatorial Forms of BmSPI38 and BmSPI39

**Figure 2:**
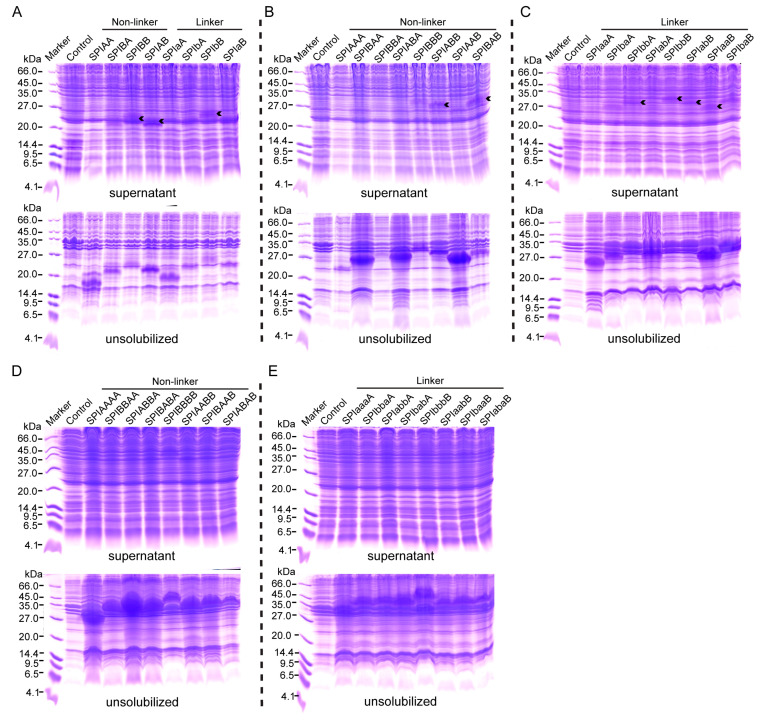
Protein expression of tandem multimers with different combinatorial forms of BmSPI38 and BmSPI39. (**A**) SDS-PAGE analysis of tandem dimer proteins. (**B**) SDS-PAGE analysis of tandem trimer proteins without linker sequence. (**C**) SDS-PAGE analysis of tandem trimer proteins with linker sequence. (**D**) SDS-PAGE analysis of tandem tetramer proteins without linker sequence. (**E**) SDS-PAGE analysis of tandem tetramer proteins with linker sequence. *E*. *coli* cells, containing the empty p28 vector, were induced with the same final concentration of IPTG as the control. The terms “supernatant” and “unsolubilized” represent the supernatant and unsolubilized fraction of the *E*. *coli* lysates, respectively. The arrows show the tandem multimer fusion proteins with different combinatorial forms of BmSPI38 and BmSPI39 expressed in *E. coli*.

In order to obtain active tandem multimer proteins with different combinatorial forms of BmSPI38 and BmSPI39, the constructed tandem multimer expression plasmids were transferred into the Origami 2(DE3) strain, and the tandem dimers, trimers, and tetramers were induced by IPTG with appropriate working concentrations. Finally, tandem dimers (Figure 2A), trimers (Figure 2B,C), and tetramers (Figure 2D,E), with different combinations expressed in *Escherichia coli*, were separated by 16.5% SDS-PAGE. The results show that the tandem multimer proteins with different combinatorial forms were mainly expressed as inclusion bodies in *E*. *coli* Origami 2(DE3) under IPTG induction. Soluble forms of dimers (SPIBA, SPIBB, SPIAB, SPIaA, SPIbA, SPIbB, and SPIaB) and trimers (SPIBBB, SPIABB, SPIBAB, SPIaaA, SPIbaA, SPIbbA, SPIabA, SPIbbB, SPIabB, SPIaaB, and SPIbaB) were also clearly detected in the supernatant of bacterial lysates. According to the results of Coomassie brilliant blue staining, the expression levels of the tandem dimers in the supernatant of bacterial lysates, from high to low, were roughly SPIBB, SPIAB, SPIbA, SPIaB > SPIaA, SPIbB, and SPIBA > SPIAA. The soluble expression levels of the tandem trimers, ranked from high to low, were approximately as follows: SPIABB, SPIBAB, SPIbbA, SPIabA, SPIbbB, SPIabB, SPIaaB, SPIbaB > SPIBBB, SPIaaA, SPIbaA > SPIAAA, SPIBAA, SPIBBA, SPIABA, and SPIAAB. Overall, the tandem multimer proteins with BmSPI39 module “B” at the carboxyl terminus had higher expression levels than that of the tandem multimers with BmSPI38 module “A” at the carboxyl terminus in the supernatant. It should be noted that although no visible expression of some combinations of tandem multimer proteins were detected in the supernatant of bacterial lysates, it cannot be ruled out that they may be expressed at a lower level in soluble form in *E*. *coli* cells.

### 2.3. Analysis of the Inhibitory Activity of Tandem Dimer Proteins Against Different Serine Proteases

**Figure 3:**
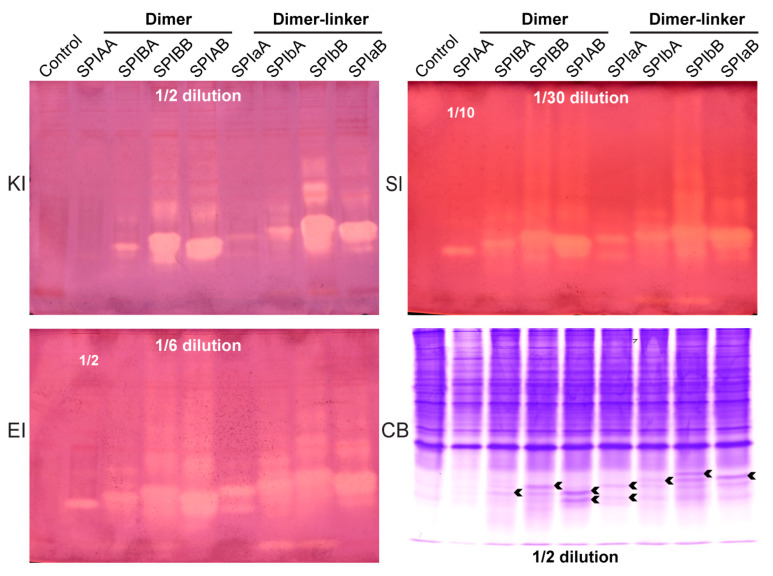
Analysis of the inhibitory activity of tandem dimers with different combinatorial forms of BmSPI38 and BmSPI39 using in-gel activity staining. “KI”, “SI”, and “EI” represent in-gel activity staining for the protease K inhibitor, subtilisin inhibitor, and elastase inhibitor, respectively. “CB” represents Coomassie brilliant blue R-250 staining under the same electrophoresis conditions. A supernatant fraction of *E*. *coli* cells transformed with the p28 plasmid was used as a negative control. It should be noted that “1/2”, “1/6”, “1/10”, and “1/30” dilution, respectively, means that the corresponding bacterial lysate supernatant was diluted 2, 6, 10, and 30 times, respectively, and then, the same volume of the protein sample was used for Native PAGE. The arrow indicates the expression bands of the fusion proteins stained with Coomassie brilliant blue R-250 corresponding to the inhibitory activity bands.

In order to investigate the effects of different combinations of BmSPI38 and BmSPI39 tandem dimerization on the total activity of their expression products in *E*. *coli* and to screen the dimerization forms with strong inhibitory activity, the activity of tandem dimerization proteins expressed in the supernatant of bacterial lysates was analyzed using the in-gel activity staining of protease inhibitors (Figure 3). Based on the expression levels of the various tandem proteins in the *E*. *coli* supernatant (Figure 3, CB) and the relative quantification range of in-gel activity assays for inhibitor activity against different proteases, the protein samples were appropriately diluted, and ultimately, the inhibitory activity of these tandem dimer proteins against protease K (Figure 3, KI), subtilisin (Figure 3, SI), and elastase (Figure 3, EI) was compared according to the strength of the inhibitory activity bands. A total of 8 forms of dimer proteins, expressed exogenously, had inhibitory activity against protease K, subtilisin, and elastase. Under the same induced expression conditions, no matter whether a linker sequence was added or not, the tandem dimer proteins with a carboxyl terminal of the BmSPI39 module “B” had a significantly stronger inhibitory effect on the above three proteases than those with a carboxyl terminal of the BmSPI38 module “A”. The inhibitory activity of dimers without a linker sequence against protease K was roughly SPIBB and SPIAB >> SPIBA > SPIAA, while the inhibitory activity of dimers with a linker sequence was roughly SPIbB and SPIaB >> SPIbA > SPIaA (Figure 3, KI). The order of inhibitory activity of tandem dimers against protease K was roughly SPIBB, SPIAB, SPIbB, SPIaB >> SPIBA, and SPIbA > SPIaA > SPIAA. The ranking of the inhibitory activity of the above 8 types of tandem dimer proteins against subtilisin (Figure 3, SI) and elastase (Figure 3, EI) was generally consistent with that of protease K. These results indicate that tandem dimerization with different combinatorial forms of BmSPI38 and BmSPI39 could affect their inhibitory activity against serine proteases. Finally, based on the strength of the inhibitory activity bands, some tandem dimerization forms with strong total activity were also screened out, such as SPIBB, SPIAB, SPIbB, and SPIaB.

### 2.4. Analysis of the Inhibitory Activity of Tandem Trimer Proteins Against Different Serine Proteases

**Figure 4:**
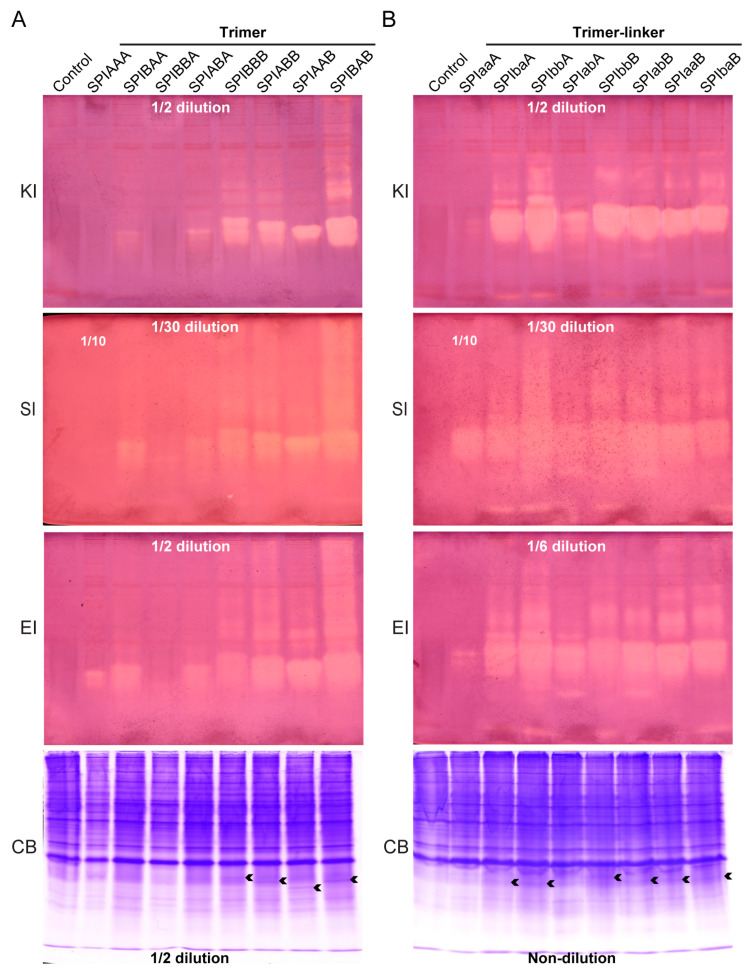
Analysis of the inhibitory activity of tandem trimers with different combinatorial forms of BmSPI38 and BmSPI39 using in-gel activity staining. (**A**) Alkaline Native PAGE analysis of BmSPI38 and BmSPI39 tandem trimer proteins without a linker sequence. (**B**) Alkaline Native PAGE analysis of BmSPI38 and BmSPI39 tandem trimer proteins with a linker sequence. “KI”, “SI”, and “EI” represent in-gel activity staining for the protease K inhibitor, subtilisin inhibitor, and elastase inhibitor, respectively. “CB” represents Coomassie brilliant blue R-250 staining under the same electrophoresis conditions. A supernatant fraction of *E*. *coli* cells transformed with the p28 plasmid was used as a negative control. It should be noted that “1/2”, “1/6”, “1/10”, and “1/30” dilution, respectively, means that the corresponding bacterial lysate supernatant was diluted 2, 6, 10, and 30 times, respectively, and then, the same volume of the protein sample was used for Native PAGE. The arrow indicates the expression bands of the fusion proteins stained with Coomassie brilliant blue R-250 corresponding to the inhibitory activity bands. “Non-dilution” indicates that the protein sample used for Native PAGE was not diluted.

To further explore the effect of the tandem trimerization of BmSPI38 and BmSPI39, with different combinatorial forms, on the total activity of their expression products in *E*. *coli*, the in-gel activity staining of protease inhibitors was used to analyze the activity of the tandem trimer proteins in the supernatant of bacterial lysates (Figure 4). The results show that in the trimers without a linker sequence, the tandem trimer proteins with the carboxyl terminal BmSPI39 module “B” had higher inhibitory activity levels against protease K than the tandem trimer proteins with the carboxyl terminal BmSPI38 module “A” (Figure 4A, KI). In the trimers with a linker sequence, the total inhibitory activity of SPIbaA, SPIbbA, SPIabA, SPIbbB, SPIabB, SPIaaB, and SPIbaB against protease K was significantly stronger than SPIaaA (Figure 4B, KI). The inhibitory activity of these tandem trimers against protease K was roughly ranked as follows: SPIbaA, SPIbbA, SPIbbB, SPIabB, SPIaaB, SPIbaB > SPIBAB > SPIBBB, SPIABB, SPIAAB, SPIabA >> SPIBAA, SPIABA, SPIaaA > SPIAAA, and SPIBBA. The inhibitory activity of the 16 tandem trimers against subtilisin (Figure 4, SI) and elastase (Figure 4, EI) was consistent with protease K. It should be pointed out that among the tandem trimer proteins (SPIBBB, SPIABB, SPIAAB, SPIBAB, SPIbbB, SPIabB, SPIaaB, and SPIbaB) with BmSPI39 module “B” at the carboxyl terminus, the trimeric forms with a linker sequence showed stronger inhibitory activity against the above three proteases than the trimeric forms without a linker sequence. These results show that tandem trimerization with different combinatorial forms of BmSPI38 and BmSPI39 was able to influence their inhibitory activity against serine proteases. Finally, some tandem trimeric forms with strong total activity were screened, such as SPIbaA, SPIbbA, SPIbbB, SPIabB, SPIaaB, and SPIbaB. Further analysis revealed that the total activity level of the above heterotrimeric forms, SPIbaA, SPIbbA, SPIabB, SPIaaB and SPIbaB, expressed in the supernatant of the bacterial lysates, was obviously higher than that of the BmSPI38 and BmSPI39 homotypic tandem trimers SPIaaA and SPIbbB. The effect of “1 + 1 > 2” was achieved.

### 2.5. Analysis of the Inhibitory Activity of Tandem Tetramer Proteins Against Different Serine Proteases

**Figure 5:**
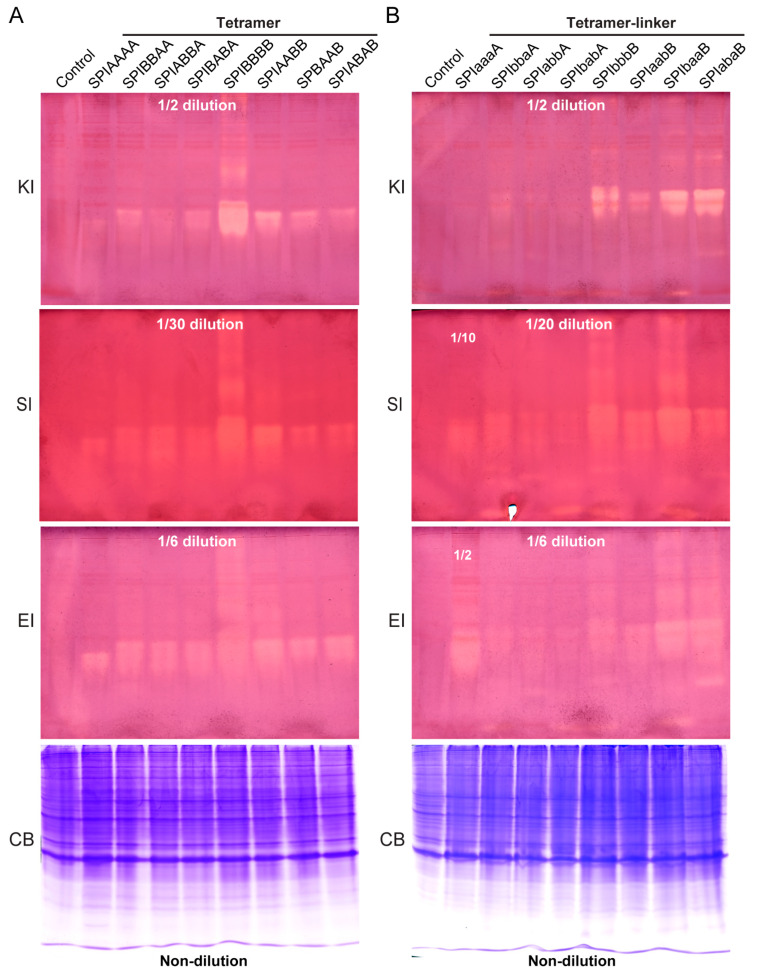
Analysis of the inhibitory activity of tandem tetramers with different combinatorial forms of BmSPI38 and BmSPI39 using in-gel activity staining. (**A**) Alkaline Native PAGE analysis of BmSPI38 and BmSPI39 tandem tetramer proteins without a linker sequence. (**B**) Alkaline Native PAGE analysis of BmSPI38 and BmSPI39 tandem tetramer proteins with a linker sequence. “KI”, “SI”, and “EI” represent in-gel activity staining for the protease K inhibitor, subtilisin inhibitor, and elastase inhibitor, respectively. “CB” represents Coomassie brilliant blue R-250 staining under the same electrophoresis conditions. A supernatant fraction of *E*. *coli* cells transformed with the p28 plasmid was used as a negative control. It should be noted that “1/2”, “1/6”, “1/10”, “1/20”, and “1/30” dilution, respectively, means that the corresponding bacterial lysate supernatant was diluted 2, 6, 10, 20, and 30 times, respectively, and then, the same volume of the protein sample was used for Native PAGE. “Non-dilution” indicates that the protein sample used for Native PAGE was not diluted.

We further evaluated the total activity of the expressed products of the different combinations of BmSPI38 and BmSPI39 in *E*. *coli* using in-gel activity staining technology of protease inhibitors in order to screen for tetramer forms with stronger inhibitory activity (Figure 5). The results show that the 16 forms of tandem tetramer proteins expressed in *E*. *coli* cells exhibited different degrees of inhibitory activity against protease K (Figure 5, KI), subtilisin (Figure 5, SI), and elastase (Figure 5, EI). Under the same cultivation conditions, among the tandem tetramer proteins without a linker sequence, SPIBBBB showed the strongest inhibitory activity against subtilisin (Figure 5A, SI), protease K (Figure 5A, KI), and elastase (Figure 5A, EI), followed by SPIAABB. Among the tetramer proteins with a linker sequence, the inhibitory ability of the tandem tetramers with BmSPI39 module “B” at the carboxyl terminus against the above three proteases (Figure 5B) was stronger than that of the tandem tetramers with BmSPI38 module “A” at the carboxyl terminus. The above 16 forms of tandem tetramers were ranked, in order of their inhibitory activity against protease K, from strong to weak, as follows: SPIBBBB >> SPIbbbB, SPIbaaB, SPIabaB > SPIAABB, SPIaabB > SPIBBAA, SPIABBA, SPIBABA, SPIBAAB, SPIABAB, SPIbbaA > SPIAAAA, SPIaaaA, SPIaabA, and SPIbabA. These results indicate that tandem tetramerization with different combinatorial forms of BmSPI38 and BmSPI39 could affect their inhibitory activity against serine proteases. Eventually, four forms of tetramer proteins with strong total activity were also screened, including SPIBBBB, SPIbbbB, SPIbaaB, and SPIabaB.

## 3. Discussion

In this study, 40 tandem multimer expression vectors with different combinatorial forms of BmSPI38 and BmSPI39 were successfully constructed by means of protein engineering. Finally, 8 active tandem dimer proteins, 16 tandem trimer proteins, and 16 tandem tetramer proteins were obtained. It was confirmed that the expression levels and total activity of BmSPI38 and BmSPI39 fusion proteins in *E*. *coli* cells can be increased by different tandem combinations, thereby achieving the effect of “1 + 1 > 2”.

Elastase is a serine protease with broad specificity, which is capable of hydrolyzing various connective tissue components, such as elastin, proteoglycans, fibronectin, and different types of collagen. Elastin is the main component of elastic fibers, which mainly exists in ligaments and vascular walls. It provides tissue elasticity and tensile strength and is an important part of tissues, such as skin, lungs, arteries, and ligaments. However, the excessive production of elastase may lead to tissue damage, which may cause or aggravate a variety of diseases, such as emphysema [25,26], pancreatitis [27], chronic bronchitis [28], nephritis [29], and autoimmune diabetes [30], seriously endangering human health. In addition, elastase can also destroy collagen fibers and tissue basement membranes, causing cancer [31,32]. Its hydrolysis of skin elastic fibers may also lead to wrinkling or relaxation of the skin, so inhibiting the activity of elastase is thought to prevent skin aging [33,34]. In this context, the study of elastase inhibitors is particularly important. They can effectively inhibit the activity of elastase and have important applicational value in the development of drugs, such as anti-inflammation, anti-tumor, and anti-skin aging drugs [35,36,37]. It has been found that the cocoon extracts of *Antheraea assamensis*, *Bombyx mori*, and *Philosamia cynthia ricini* have a certain inhibitory effect against porcine pancreatic elastase [37]. Other studies have shown that silk contains abundant protease inhibitors, and TIL protease inhibitors, represented by BmSPI38 and BmSPI39, enter the cocoon layer during silk secretion, causing cocoons to have strong antimicrobial protease hydrolysis characteristics [18,19,38,39]. Previous studies have confirmed, for the first time, that BmSPI38 and BmSPI39 can strongly inhibit the activity of porcine pancreatic elastase and have clarified the inhibitory effects of silkworm protease inhibitors on elastase [40]. In this study, two functionally similar protease inhibitors, BmSPI38 and BmSPI39, were expressed with different combinations in tandem. Compared with the BmSPI38 and BmSPI39 homotypic multimers, some heterotypic multimers had stronger inhibitory activity against elastase, such as SPIAB and SPIaB (Figure 3, EI). As a powerful inhibitor of elastase, the tandem expression of BmSPI38 and BmSPI39 not only provides a powerful tool for studying the relationship between the structure and function of elastase but also has important research value in biomedicine and skin care fields. However, as a potential template for anti-inflammatory and anti-aging lead drugs, it is critical to perform in vivo evaluations to verify their effectiveness and safety in practical applications.

Multimerization of protease inhibitors is a common phenomenon, and it plays an important role in regulating the advanced structure and biological activity of protease inhibitors. Cystatin C, a cysteine protease inhibitor, plays a crucial role in various physiological and pathological processes, such as vascular remodeling and inflammation. Its activity can be regulated by altering its oligomerization state [41,42]. DM43 is a homodimeric metalloproteinase inhibitor isolated from the serum of *Didelphis marsupialis*. It binds to the metalloproteinase Jarararhagin in the venom of *Bothrops jararaca* by non-covalent binding, thereby effectively neutralizing its toxicity [43]. Our previous research found that the serine protease inhibitors BmSPI38 and BmSPI39 of silkworm are able to effectively defend against the invasion of pathogenic fungi into silkworm by inhibiting the activity of virulence proteases secreted by the fungus [16,17]. Further studies have found that BmSPI38 and BmSPI39 exist and function mainly in the form of tetramers in various tissues of silkworm [20].

Studies have shown that gene tandem expression technology can not only effectively improve the expression levels and structural stability of small molecule recombinant proteins but can also shield the damage of toxic proteins to the host, so it has been widely used in the expression of exogenous proteins [44,45,46,47,48,49]. Polypeptide antibiotics are small peptides encoded by the genome DNA of organisms and play an important role in host defense. Researchers designed a tandem repeat multimer of the polypeptide antibiotic hPAB-β and constructed recombinant plasmids containing 1 to 8 copies of the hPAB-β gene. The active tandem repeat multimers hPAB-β were successfully expressed in *E*. *coli*, which laid a foundation for their large-scale preparation [50]. Thymosins are polypeptide hormones secreted by the animal or human thymus, among which thymosin α1 (Tα1) has the strongest biological activity; can stimulate the T cell maturation and expression of interleukin-2, the interleukin-2 receptor, and cell adhesion molecule differentiation cluster 2; and can inhibit hepatitis B virus replication in HepG2 tumor cells. Researchers have successfully expressed the 4×Tα1 tandem protein in *E*. *coli* M15 by the tandem method. This tandem protein not only exhibits good biological activity but has also been proven to effectively stimulate the proliferation of spleen lymphocytes of mice, demonstrating the potential of tandem proteins in enhancing biological activity [51]. The fusion protein was expressed in a tandem form by fusing four proteins: the protein transduction domain, human serum albumin (HSA), a linker peptide, and a sleep peptide. The fusion proteins obtained not only retained the activity of the sleep peptide but also overcame the difficulties of clinical applications due to their small molecular weight and short half-life. The novel fusion protein dTMP-has, formed by the fusion of a thrombopoietin mimetic peptide dimer (dTMP) and has, showed better bioactivity in vitro and in vivo [52,53]. It was found that both the serine protease inhibitors BmSPI38 and BmSPI39 of silkworm possess a TIL (trypsin inhibitor-like cysteine-rich) domain containing eight conserved cysteine residues. Homotypic tandem expression, based on genetic engineering technology, can significantly enhance the inhibitory activity of BmSPI38 and BmSPI39 against subtilisin, protease K, and elastase [21,22]. This study, for the first time, adopted the concept of protein design to successfully achieve the heterologous expression of BmSPI38 and BmSPI39 dimer (Figure 2A), trimer (Figure 2B,C), and tetramer proteins (Figure 2D,E) in *E*. *coli* by linking two protease inhibitors with the same domain (TIL) in different combinations. Under the same induction conditions, different combinatorial forms of tandem multimerization can significantly increase the expression levels of BmSPI38 and BmSPI39 in the supernatant of *E*. *coli* Origami 2(DE3) cells compared to combinatorial forms of homotypic tandems.

To accurately evaluate the total activity of tandem multimers with different forms of BmSPI38 and BmSPI39 in *E*. *coli* and to screen out the tandem multimers with strong inhibitory activity, Native PAGE and in-gel activity staining of protease inhibitors were used to compare the inhibitory activity of the inhibitors against three different serine proteases. Compared with traditional detection technology, this technology can not only maintain the natural conformation of the protein but also does not need to purify the protease inhibitors from the tissue samples, thus avoiding the problems of denaturation and degradation and loss caused by protein purification and other operations. This technique is highly specific and sensitive and can be used to comprehensively, efficiently, and visually evaluate the inhibitory ability of the inhibitor against the specified serine protease by comparing the strength of the inhibitory activity bands in the gel [54]. Previous studies have confirmed that BmSPI38 and BmSPI39 have significant inhibitory effects against subtilisin, protease K, and elastase [16,40]. This study found that under the same induction conditions, the level of inhibitory activity of the tandem dimers with BmSPI39 module “B” at the carboxyl terminus on protease K (Figure 3, KI), subtilisin (Figure 3, SI), and elastase (Figure 3, EI) was significantly higher than that of the dimers with BmSPI38 module “A” at the carboxyl terminus. Similar trends were also observed in the tandem trimers without a linker sequence (Figure 4A, EI, SI, and KI). In addition, the expression levels of tandem dimers (Figure 3, CB) and trimers (Figure 4A, CB) with BmSPI39 module “B” at the carboxyl terminus were higher in the supernatant of *E*. *coli*, which was generally consistent with the results of SDS-PAGE (Figure 2A–C). This may be due to the fact that the linking order between the BmSPI38 and BmSPI39 modules affects the formation of an advanced structure and the expression levels of the proteins, thus affecting the total activity of the tandem multimers in *E*. *coli* cells. Similar studies have also shown that the linking order between protein modules significantly influences the activity of their fusion proteins. It was found that the xylanase activity of the fusion enzyme SWOI-XYNII, obtained by connecting the protein SWOI with the xylanase XYNII using genetic engineering technology, was enhanced, while the xylanase activity of XYNII-SWOI was reduced [55]. At present, there is still a lack of advanced structural data on the binding of BmSPI38 and BmSPI39 to proteases, which limits the in-depth understanding of their mechanisms of action. Therefore, the specific mechanism of action of BmSPI38 and BmSPI39 tandem multimers against different proteases still needs to be further investigated. These studies not only help to reveal the molecular basis of BmSPI38’s and BmSPI39’s inhibitory activity but may also provide important clues for the development of novel protease inhibitors.

In order to improve the expression levels and activity of exogenous proteins and reduce production costs, researchers usually fuse target proteins with larger soluble protein tags, such as maltose-binding proteins [56]. However, due to the large molecular weight of such tag proteins, they may interfere with the correct formation of the advanced structure of the target protein. Adding a small molecular weight flexible linker peptide between two domains has become a convenient and efficient protein modification strategy, which does not require a comprehensive understanding of the structural information of the target protein. Flexible linker peptides, such as (GGGGS)n, which do not have the ability to form a specific secondary structure, generally exist in the form of random coils in space. (GGGGS)n can increase protein solubility, resist proteolytic degradation, and provide the flexible space required for the realization of protein functions, so that each domain does not interfere with each other, so it has been widely used [57,58]. In this study, it was found that in the trimer proteins (SPIBBB, SPIABB, SPIAAB, SPIBAB, SPIbbB, SPIabB, SPIaaB, and SPIbaB) with BmSPI39 module “B” at the carboxyl end of the tandem, the total activity of the trimers with a flexible linker peptide was generally stronger than that of the trimers without a linker peptide (Figure 4A,B). Studies have shown that the length of the linker peptide between fusion proteins significantly affects the expression and activity of alkaline pectinase fusion enzymes and the small molecule hapten. The longer the linker peptide, the easier it is for the protein to fold correctly and exert activity [59,60]. Additionally, codon optimization of the (GGGGS)_n_ linker peptide can also substantially increase the expression and activity of recombinant fusion antibodies in *E*. *coli* [61].

Previous studies have found that homotypic multimerization of BmSPI38 and BmSPI39 can enhance their inhibitory activity against serine proteases [21,22]. In this study, the inhibitory activity of protease K was compared vertically between BmSPI38 and BmSPI39 tandem dimers (Figure 3, KI), trimers (Figure 4, KI), and tetramers (Figure 5, KI), and it was found that the activity level of tandem trimers with flexible linking peptides was much higher than that of other combinatorial forms as a whole. Finally, a batch of multimer forms with stronger total activity were screened, such as SPIbaA, SPIbbA, SPIbbB, SPIabB, SPIaaB, and SPIbaB. Further analysis revealed that the total activity of the SPIbaA, SPIbbA, SPIabB, SPIaaB, and SPIbaB expressed in the *E. coli* supernatant was superior to that of the homotypic multimers of BmSPI38 and BmSPI39 (SPIaaA and SPIbbB), confirming that tandem multimerization of different combinations was an effective way to enhance their total activity.

## 4. Materials and Methods

### 4.1. Escherichia coli Strain and Reagents

*TransStart*^®^ *TopTaq* DNA polymerase was purchased from TransGen Biotechnology (Beijing, China). *Nde* I, *Not* I, *Bam*H I, and *Bgl* II restriction endonucleases were purchased from Takara (Dalian, China). Subtilisin A, derived from *Bacillus licheniformis*, N-acetyl-D,L-phenylalanine-β-naphthylester, and Fast Blue B Salt, were purchased from Sigma-Aldrich (St. Louis, MO, USA). Protease K, derived from *Tritirachium album limber*, was purchased from Roche (Mannheim, Germany). Elastase from a porcine pancreas was purchased from BBI Life Sciences (Shanghai, China). The p28 expression plasmid and *E*. *coli* Origami 2(DE3) strain were both preserved by the College of Biological Science and Engineering, Shaanxi University of Technology.

### 4.2. Construction of Tandem Multimer Expression Vectors with Different Combinations of BmSPI38 and BmSPI39

The basic unit vectors *Nde* I/*Bam*H I-*SPI38*-*Bgl* II-TA (4029 bp), *Nde* I/*Bam*H I-*SPI38L*-*Bgl* II (4074 bp), *Nde* I/*Bam*H I-*SPI39*-*Bgl* II, *Nde* I/*Bam*H I-*SPI39L*-*Bgl* II; the His_6_-*BmSPI38*-monomer vectors; and His_6_-*BmSPI39*-monomer vectors used in this study were successfully constructed in the early stage [21,22]. Firstly, the coding frame sequence of BmSPI38 with the signal peptide removed (*SPI38*) and the coding frame sequence of BmSPI38 containing a termination codon (*SPI38end*) were named “A”, and the coding frame sequence of BmSPI39 with the signal peptide removed (*SPI39*) and that containing a termination codon (*SPI39end*) were named “B”. The BmSPI38 (*SPI38-L*) and BmSPI39 (*SPI39-L*) coding frame sequences with a flexible linker sequence at the carboxyl end were named “a” and “b”, respectively. Subsequently, “A” (*SPI38end*) and “B” (*SPI39end*) were designed at the 3′ end of the fusion gene expression vector, respectively, and were connected in tandem at its 5′ end. According to our previously reported methods [21,22], *Bam*H I (G/GATCC) and *Bgl* II (A/GATCT) were a pair of isocaudarners. The His_6_-*BmSPI38*-monomer and His_6_-*BmSPI39*-monomer expression vectors were double-digested using *Nde* I and *Bam*H I. *Nde* I and *Bgl* II were then used for the double digestion of the basic unit vectors *Nde* I/*Bam*H I-*SPI38*-*Bgl* II-TA, *Nde* I/*Bam*H I-*SPI38L*-*Bgl* II-TA, *Nde* I/*Bam*H I-*SPI39*-*Bgl* II-TA, and *Nde* I/*Bam*H I-*SPI39L*-*Bgl* II-TA. Then, the recovered *Nde* I/*Bam*H I-*SPI38*-*Bgl* II fragment, *Nde* I/*Bam*H I-*SPI38L*-*Bgl* II fragment, *Nde* I/*Bam*H I-*SPI39*-*Bgl* II fragment, and *Nde* I/*Bam*H I-*SPI39L*-*Bgl* II fragment were ligated with the His_6_-*BmSPI38*-momomer and His_6_-*SPI39*-monomer vector fragment, respectively. Finally, 8 forms of tandem dimer expression vectors were obtained and were named *SPIAA*, *SPIBA*, *SPIBB*, *SPIAB*, *SPIaA*, *SPIbA*, *SPIbB*, and *SPIaB*.

Similarly, the tandem dimer expression vectors *SPIAA*, *SPIBA*, *SPIBB*, *SPIAB*, *SPIaA*, *SPIbA*, *SPIbB*, and *SPIaB* were double-digested using *Nde* I and *Bam*H I. Then, *Nde* I and *Bgl* II were used to double digest the basic unit vectors *Nde* I/*Bam*H I-*SPI38*-*Bgl* II, *Nde* I/*Bam*H I-*SPI38L*-*Bgl* II, *Nde* I/*Bam*H I-*SPI39*-*Bgl* II, and *Nde* I/*Bam*H I-*SPI39L*-*Bgl* II. Thus, “A” (*SPI38*) and “B” (*SPI39*) could be connected to the 5′ end of *SPIAA*, *SPIBA*, *SPIBB* and *SPIAB*, respectively, so as to construct *SPIAAA*, *SPIBAA*, *SPIBBA*, *SPIABA*, *SPIBBB*, *SPIABB*, *SPIAAB*, and *SPIBAB*. Meanwhile, the 5′ end of *SPIaA*, *SPIbA*, *SPIbB*, and *SPIaB* could be connected in tandem with “a” (*SPI38-L*) and “b” (*SPI39-L*) to construct *SPIaaA*, *SPIbaA*, *SPIbbA*, *SPIabA*, *SPIbbB*, *SPIabB*, *SPIaaB*, and *SPIbaB*, thus completing the construction of 16 trimer expression vectors. Next, 16 tetramer expression vectors were constructed: *SPIAAAA*, *SPIBBAA*, *SPIABBA*, *SPIBABA*, *SPIBBBB*, *SPIAABB*, *SPIBAAB*, *SPIABAB*, *SPIaaaA*, *SPIbbaA*, *SPIabbA*, *SPIbabA*, *SPIbbbB*, *SPIaabB*, *SPIbaaB*, and *SPIabaB*. Finally, the successfully constructed tandem dimer, trimer, and tetramer expression plasmids with different combinatorial forms of BmSPI38 and BmSPI39 were double-digested using *Nde* I and *Not* I and were sent to a company for sequencing verification.

### 4.3. Prokaryotic Expression of Tandem Multimer Proteins with Different Combinatorial Forms of BmSPI38 and BmSPI39

The successfully constructed tandem multimer expression plasmids with different combinatorial forms of BmSPI38 and BmSPI39 were transformed into the *E*. *coli* Origami 2(DE3) strain. When the *OD*_600_ of the bacteria was 0.6–1.0, under the culture conditions of 37 °C and 220 r/min, IPTG was added to the tandem dimers at a final concentration of 0.1 mmol/L and to the tandem trimers and tetramers at a final concentration of 0.02 mmol/L, and then, the expression was induced at 16 °C for 20 h. After centrifugation at 6000 r/min for 30 min, the cells were collected and resuspended with a 1× binding buffer (20 mmol/L of Tris-HCl, 500 mmol/L of NaCl, and pH 7.9). After ultrasonic crushing and centrifugation, the bacterial supernatant was collected. The protein samples were mixed with a 5× SDS-PAGE loading buffer (1 mol/L of Tris-HCl, pH 6.8, 10% SDS, 25% glycerol, and 0.5% bromophenol blue) at a volume ratio of 4:1; were boiled for 10 min; then, were separated by 16.5% SDS-PAGE; and finally, were stained with Coomassie brilliant blue R-250.

### 4.4. Native PAGE

Following the same method as in previous reports [20], the proteins were separated on 10% alkaline Native PAGE gels in a Tris-Glycine electrophoresis buffer at pH 8.3. Based on the relative quantification range of in-gel activity assays for inhibitor activity against different proteases, the protein samples were appropriately diluted and then mixed with a 4× Native PAGE loading buffer (40 mmo/L of Tris-HCl, pH 8.0, 40% glycerol, and 0.032% bromophenol blue) in a volume ratio of 3:1 and were subjected to electrophoresis at 4 °C. The gel, after electrophoresis, could be used for the subsequent in-gel activity staining of protease inhibitors and Coomassie brilliant blue R-250 staining.

### 4.5. In-Gel Activity Staining of Protease Inhibitors

The in-gel activity staining of protease inhibitors was performed in reference to a previously reported method [40]. The gels, after electrophoresis, were placed in 0.07 mg/mL of subtilisin, 0.07 mg/mL of protease K, and 5 mg/mL of the elastase solution, respectively, and incubated at 37 °C, at 45 r/min, for 30 min in the dark. The protease solution was recovered, and the gel surface was washed with ddH_2_O then placed in the dark for 30 min at 37 °C. According to the volume ratio of 1:10, a mixture of a matrix solution (200 mg of N-acetyl-D,L-phenylalanine-β-naphthyl ester dissolved in 100 mL of N,N′-dimethylformamide) and a staining solution (100 mg of Fast Blue B Salt, dissolved in 100 mL of 100 mmol/L of a Tris-HCl buffer containing 20 mmol/L of CaCl_2_ at pH 8.0) was added and then incubated under the conditions of 37 °C and 45 r/min with shaking in the dark for 15 min. Next, the staining solution was discarded, and then, the gel surface was washed by adding ddH_2_O to terminate the reaction. The principle of staining is as follows: the protease on the gel can decompose the chromogenic substrate N-acetyl-D,L-phenylalanine-β-naphthyl ester, and the generated β-naphthol can dye the gel to purplish red by a diazo coupling reaction [62,63]. If the in-gel protease inhibitor can inhibit the corresponding protease activity, its location will not be stained and will appear as a white band.

## 5. Conclusions

This study successfully achieved the expression of tandem multimer proteins with different combinations of the silkworm protease inhibitors BmSPI38 and BmSPI39 in *E*. *coli*. It was confirmed that tandem multimerization of different combinations was an effective method to improve the total activity and expression levels of the BmSPI38 and BmSPI39 fusion proteins, and some tandem multimer proteins with strong total activity were successfully screened, for example, SPIbaA, SPIbbA, SPIbbB, SPIabB, SPIaaB, and SPIbaB. This study not only lays a foundation for the exogenous production and development of BmSPI38 and BmSPI39 but also provides a reference for the construction of tandem and multimerization exploration of other protease inhibitors.

## Data Availability

Data are contained within the article.
